# Clinical Competence of Nurses and the Associated Factors in Public Hospitals of Gamo Zone, Southern Ethiopia: A Cross-Sectional Study

**DOI:** 10.1155/2023/9656636

**Published:** 2023-09-26

**Authors:** Shitaye Shibiru, Zeleke Aschalew, Mekidim Kassa, Agegnehu Bante, Abera Mersha

**Affiliations:** ^1^School of Nursing, College of Medicine and Health Sciences, Arba Minch University, Arba Minch, Ethiopia; ^2^School of Public Health, College of Medicine and Health Sciences, Arba Minch University, Arba Minch, Ethiopia

## Abstract

**Introduction:**

Nursing competency is an essential component for improving the quality of care in the healthcare system. However, assessing competency solely on the dimensions of skills and knowledge does not provide complete picture of a nurse ability to provide quality patient care. This is because it lacks focus on the nurse's attitudes and values, which are also important determinants of clinical competence. Therefore, this study aimed to assess the comprehensive clinical competence of nurses and its associated factors in public hospitals of Gamo Zone, Southern Ethiopia.

**Materials and Methods:**

A cross-sectional study was conducted using a census method to collect information from nurses through self-administered questionnaires. The data were entered into EpiData version 3.1 and exported to Stata version 15 for analysis. A linear regression model was used to identify factors associated with clinical competence.

**Results:**

In this study, the average clinical competence of nurses was 177.32, with a standard deviation of 19.19, and 31.2% of the respondents had a high level of clinical competence. Associated factors identified with clinical competence include gender, age, marital status, qualification, position, work experience, unit, interest in their profession, critical thinking disposition, clinical self-efficacy, and emotional intelligence.

**Conclusions:**

The overall level of clinical competence among nurses in this study was moderate. As such, nurses improve their clinical competence by receiving training and development opportunities that focus on critical thinking, clinical self-efficacy, and emotional intelligence; working in a supportive work environment that encourages them to take risks and learn from their mistakes; and being monitored and coached on a regular basis.

## 1. Introduction

Clinical competence in nursing refers to the knowledge, skills, attitudes, and values that nurses need to provide safe and effective care to patients [1, 2]. It is essential for improving the quality of care, increasing patient satisfaction, and promoting nursing as a profession [1, 3].

Nurses play a vital role in achieving the Sustainable Development Goal (SDG) 3 of reducing maternal and neonatal mortality by 2030. They are part of an integrated team of professionals in maternal and newborn health (MNH), and their unique skills and knowledge are essential for providing high-quality care to mothers and newborns [4, 5]. The United Nations International Children's Emergency Fund (UNICEF) and the World Health Organization (WHO) recognize the importance of nurses in MNH. They have stated that nurses are “instrumental in influencing patient outcomes” [5]. This is because nurses have a deep understanding of the needs of mothers and newborns, and they are able to provide individualized care that is tailored to each patient's specific situation [4]. In addition to their technical skills, nurses also bring a set of core values to their work. These values include compassion, respect, and commitment to quality care [6]. Nurses are dedicated to providing the best possible care to their patients, and they are always striving to improve their skills and knowledge [4]. By working together with other healthcare professionals, nurses can make a real difference in the lives of mothers and newborns. They can help to reduce maternal and neonatal mortality, and they can ensure that all mothers and newborns have access to the care they need [4, 5].

Continuing competency is essential for nurses to maintain their professional growth, confidence in the workplace, and ability to provide safe and positive care to patients. Nurses with higher clinical competency are more likely to establish empathetic relationships with patients and utilize their skills effectively in clinical settings [7–9]. On the other hand, burnout is negatively associated with clinical competency, meaning that nurses who are more burned out are less likely to be clinically competent [10].

Studies have shown that nurses' clinical competence varies depending on the setting and the specific components of competence being assessed. For example, a study in Iran found that nurses had the highest level of clinical competency in knowledge and the lowest level in skills [11]. However, a study in China found that nurses had the highest level of clinical competency in attitudes and the lowest level in practice [12]. The mean score of nurses' clinical competence has also been shown to vary depending on the setting. For example, a study of nurses in critical care settings found that the mean score was 174.4, while a study of nurses in primary care settings found that the mean score was 2.7 [13, 14]. In pocket studies, which are small-scale studies that are conducted in a clinical setting, the mean scores of nurses' clinical competence have been shown to be above average [15–17]. For example, a pocket study of nurses in emergency departments found that the mean score was 104.2 [18]. The components of clinical competence that have been studied most often include knowledge, skills, attitudes, and practice. However, other components of clinical competence have also been studied, such as quality assurance [19], job responsibility [20], caring ability [21], and research ability [22]. Other components of clinical competence include managing situations and work [15], critical thinking, and research attitude [23].

Nurses' clinical competence is influenced by a variety of factors, including their background, training, and experience [24, 25]. Other factors that have been associated with nurses' clinical competence include attitudes, personality, evaluation of the quality of nursing care [26], emotional intelligence, self-efficacy [27], critical thinking [28], job satisfaction [29], the autonomy of nurses [25], and prudence [28]. Studies conducted in Korea and Iran have also found that nurses with high compassion satisfaction (CS) and low compassion fatigue (CF) (burnout (BO) and secondary traumatic stress) tend to have higher clinical competence [24, 30]. Raising nurses' professional quality of life (ProQOL) levels can also help improve their clinical competence [24].

Assessing clinical competence solely on the basis of skills and knowledge does not provide a comprehensive picture of a nurse's ability to deliver quality patient care [24]. Nurses' clinical competence is a key component of patient care that requires regular and thorough evaluation to ensure the best possible outcomes for patients. However, this area is often overlooked, and nurses' competence does not receive the recognition it deserves in the healthcare system. Therefore, this study aimed to assess the comprehensive clinical competence of nurses and its associated factors in public hospitals of Gamo Zone, Southern Ethiopia.

## 2. Materials and Methods

### 2.1. Study Design and Period of the Study

The study adopted a cross-sectional study design and was conducted in public hospitals in Gamo Zone, Southern Ethiopia, from January 1 to 20, 2022.

### 2.2. Study Setting

Gamo Zone is an administrative zone in the Southern Ethiopia. Gamo Zone has one general hospital and five primary hospitals which include Arba Minch General Hospital, Dilfana Primary Hospital, Chencha Primary Hospital, Kamba Primary Hospital, Gerese Primary Hospital, and Selamber Primary Hospital.

### 2.3. Study Population

Nurses working in a neonatal intensive care unit, operating room, pediatric, surgical, and medical wards, and emergency units were chosen for the study.

### 2.4. Eligibility

All nurses who completed the probation period in each hospital were recruited in this study, and those on annual leave at the time of data collection were excluded.

### 2.5. Sample Size Determination and Sampling Procedure

Cochran's formula was used to determine the sample size. The assumptions were mean score and standard deviation (2.82 ± 0.53) of nurses' clinical competence from the study conducted in Iran among 230 nurses [25], a 95% level of confidence, and a 15% nonresponse rate. Based on the stated assumptions, the calculated sample size for this study was 359. Nevertheless, to increase the power and precision of the study, all the nurses who worked in the respective wards in each hospital were involved. Census method was used to involve study participants in this study.

### 2.6. Data Collection Tool

A structured self-administered survey tool was adapted from different works of literature and used to collect the data. The tool consists of eight parts that include the following:

- Socio-demographic and professional characteristics.
- Clinical competence was assessed using the Competency Inventory for Registered Nurses (CIRN). The CIRN is a 55-item tool with seven dimensions: clinical care, leadership, interpersonal relations, legal/ethical practice, professional development, teaching/coaching, and research aptitude/critical thinking. It was scored by using the five-point Likert scale ranging from 0 to 4 and the highest score represented better clinical competence for each subdimension [24, 31, 32].
- Practice environment was measured with the Nursing Work Index-Revised (NWI-R) which contains 12 items composed of three dimensions. It was measured by using a 4-point Likert scale and the higher score represented that the nursing practice environment tends to be autonomous, supportive, and collaborative [32–35].
- Critical thinking disposition was measured using a 35-item tool with eight dimensions. It was measured by using a 5-point Likert scale and the higher score represented a higher critical thinking disposition [32, 36].
- Professional quality of life was measured using a 30-item tool with three dimensions. Each dimension was measured by using a 5-point Likert scale and the higher score represented a higher professional quality of life [37].
- Clinical self-efficacy was measured using the Self-Efficacy in Clinical Performance (SECP) questionnaire. The SECP is a 37-item tool with five dimensions. It was measured by using a 4-point Likert scale and the higher score represented good clinical self-efficacy [38–40].
- Personality traits were measured using the 10-item version of the Big Five-Factor Inventory. It was measured by using a 5-point Likert scale and the higher score represented good personality traits [41–44].
- Emotional intelligence was measured using the Schutte Self Report Emotional Intelligence Test (SSEIT-33). The SSEIT-33 is a 33-item tool with three dimensions and additional uncategorized groups. It was measured by using a 5-point Likert scale and the higher score represented good emotional intelligence [44–46].

The data were collected by twelve data collectors who had bachelor's degree in nursing and were supervised by four supervisors who had a master's degree in nursing. Both the data collectors and supervisors were trained before starting data collection. The data collectors were given information about the study's aim before providing the self-administered questionnaire to the study participants. The questionnaire was offered to the nurses when they were not busy with patient management and comfortable in a private room to fill it out freely.

### 2.7. Study Variables

The dependent variable was the nurses' clinical competence. Background and professional-related characteristics, practice environment, critical thinking, professional quality of life, personality and emotional intelligence, and clinical self-efficacy were the independent variables for this study.

### 2.8. Data Quality Control

To ensure the consistency and standardization of data collection techniques, the data collection tool was pretested and the data collectors received extensive training. The content, face, and construct validity of the tool were checked, and the interclass correlation coefficients (ICC) of all the tools were greater than 0.7 to ensure the reliability.

### 2.9. Data Processing and Analysis

The data were coded, cleaned, edited, and entered into EpiData version 3.1. They were then exported to Stata version 15 for analysis. Univariate analysis was performed to calculate proportions, percentages, and summary statistics. The normality distribution was examined using a scatter plot, kurtosis, and skewness. A multiple linear regression model was used to identify factors associated with clinical competence. All variables with a *P* value less than 0.25 in the simple linear regression and variables that were significant in previous studies were included in the final model to control for all possible confounders. The goodness of fit was tested using the *R*-squared (*R*^2^) value. A multicollinearity test was performed to check for correlation between the independent variables. A variance inflation factor (VIF) of greater than 10 and a tolerance value of less than 0.1 were considered to be indicative of multicollinearity. A crude and adjusted beta (*β*) coefficient with a 95% confidence interval (CI) was estimated to identify the factors affecting nurses' clinical competence. In this study, a *P* value less than 0.05 was considered to be statistically significant. The results were presented using simple frequencies, summary measures, tables, and figures.

## 3. Results

### 3.1. Background and Professional Characteristics

In this study, 404 nurses were involved, and the mean and standard deviation of the age were 33.39 ± 6.34. Two hundred and sixty one (64.6%) were females and 240 (59.4%) were Orthodox religion followers. Of the participants, 260 (64.4%) were married and 252 (62.4%) were qualified for BSc. Regarding job title/position, 375 (92.8%) were staff nurses, 18 (4.5%) had focal persons, and 11 (2.7%) had nursing managers/directors (Table 1).

### 3.2. Practice Environment

The mean and standard deviation of the overall practice environment among nurses in this study was 32.94 ± 10.07. Regarding dimensions, the adequate staffing and resources dimension had a mean and standard deviation of 11.50 ± 3.97, autonomy 13.58 ± 4.40, and nurse-physician collaboration 7.86 ± 2.89.

### 3.3. Critical Thinking Disposition

**Figure 1:**
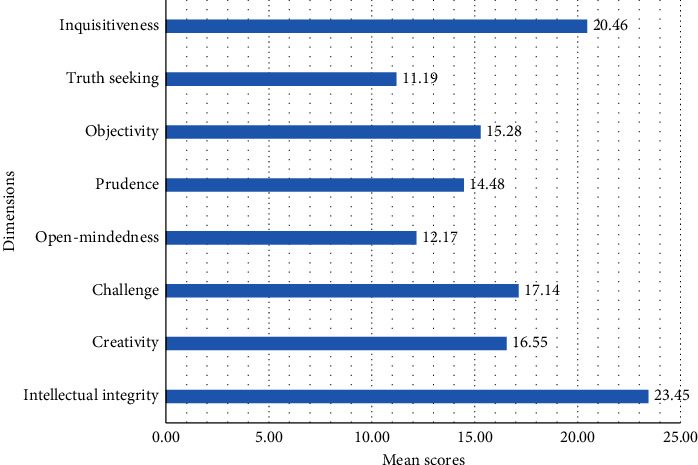
Mean scores for the dimensions of the critical thinking disposition in hospitals of Gamo Zone, Southern Ethiopia, 2022 (*n* *=* 404).

This study reported the mean and standard deviation of the critical thinking disposition was 130.71 ± 14.25. Of the dimensions, intellectual integrity had a mean and standard deviation of 23.45 ± 3.71 (Figure 1).

### 3.4. Professional Quality of Life

The mean and standard deviation of professional quality of life was 111.09 ± 15.82. Out of the dimensions, compassion satisfaction had a mean and standard deviation of 42.10 ± 5.34, burnout had 36.49 ± 7.43, and secondary traumatic stress had 32.51 ± 8.99.

### 3.5. Clinical Self-Efficacy

**Figure 2:**
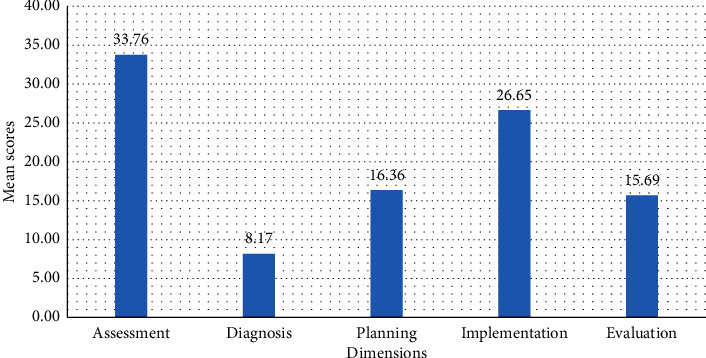
Mean scores for the dimensions of clinical self-efficacy in hospitals of Gamo Zone, Southern Ethiopia, 2022 (*n* *=* 404).

Findings from this study indicated the mean and standard deviation of the overall clinical self-efficacy was 100.62 ± 11.37. Of the dimensions, the assessment had a mean and standard deviation of 33.76 ± 3.44 (Figure 2).

### 3.6. Personality Traits and Emotional Intelligence

The mean and standard deviations of personal traits and emotional intelligence were 34.94 ± 5.2 and 131.89 ± 8.59.

### 3.7. Clinical Competence of Nurses

**Figure 3:**
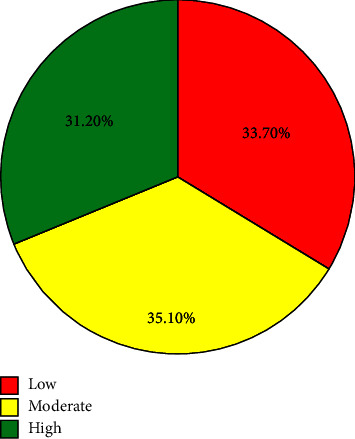
Level of clinical competence of the nurses in hospitals of Gamo Zone, Southern Ethiopia, 2022 (*n* = 404).

**Figure 4:**
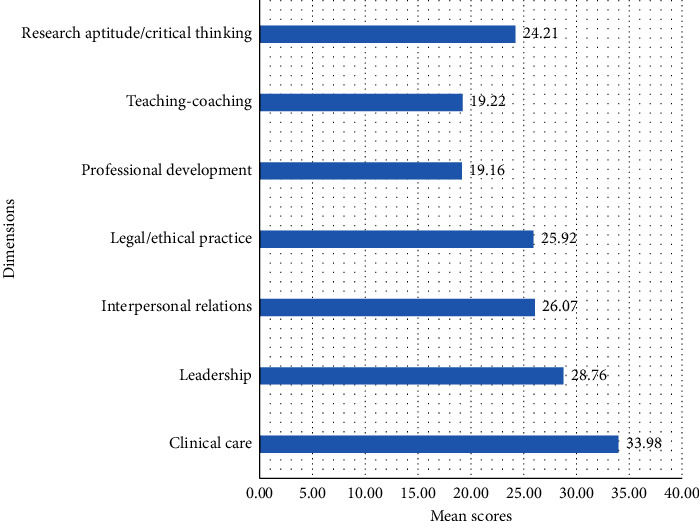
Mean scores for the dimensions of clinical competence of the nurses in hospitals of Gamo Zone, Southern Ethiopia, 2022 (*n* *=* 404).

Findings from this study indicated the mean and standard deviation of the overall clinical competence of nurses was 177.32 ± 19.19. One hundred and twenty six (31.2%) had high level of clinical competence (Figure 3). Of the dimensions, clinical care had a mean and standard deviation of 33.98 ± 4.7 (Figure 4).

### 3.8. Factors Associated with the Clinical Competence of Nurses

#### 3.8.1. Simple Linear Regression

In the bivariate model, gender, age, qualification, position/title, ward/unit, training in nursing care, interest in the nursing profession, practice environment, critical thinking disposition, professional quality of life, clinical self-efficacy, personal traits, and emotional intelligence were significantly associated with clinical competence of nurses. Fifteen variables were inputted in the bivariate model and candidates for the final model (Table 2).

#### 3.8.2. Multiple Linear Regression

In this study, gender, age, marital status, educational status, position, work experience, ward, interest in the nursing profession, critical thinking disposition, clinical self-efficacy, and emotional intelligence were significantly associated with the clinical competence of the nurses in the final model.

Being male increased the clinical competence of the nurse by 0.19 units (*β* = 0.19, 95% CI: 0.09, 0.28), and a unit increase in age of the participant decreased the clinical competence of the nurses by 0.66 units (*β* = −0.66, 95% CI: −0.81, −0.51). The clinical competence of the nurses decreased by 0.31 units (*β* = −0.31, 95% CI: −0.54, −0.08) for being a single, increased by 0.26 units (*β* = 0.26, 95% CI: 0.18, 0.33) for the qualification of the BSc, and increased by 0.12 units (*β* = 0.12, 95% CI: 0.05, 0.19) for being a ward head/focal person. A unit increase in work experience increased the clinical competence of the nurses by 0.43 units (*β* = 0.43, 95% CI: 0.29, 0.56), and working in the medical ward and emergency unit decreased the clinical competence by 0.29 (*β* = −0.29, 95% CI: −0.39, −0.18) and 0.15 (*β* = −0.15, 95% CI: −0.24, −0.06) units, respectively. A unit increase in the interest of the nurses in profession fairly increased the clinical competence of the nurses by 0.25 (*β* = 0.25, 95% CI: 0.13, 0.37) and 0.17 (*β* = 0.17, 95% CI: 0.05, 0.28) units, and a unit increase in critical thinking disposition increased the clinical competence of the nurses by 0.35 units (*β* = 0.35, 95% CI: 0.27, 0.44). The clinical competence of the nurses increased by 0.11 units (*β* = 0.11, 95% CI: 0.03, 0.19) for a unit increase in clinical self-efficacy and by 0.14 units (*β* = 0.14, 95% CI: 0.06, 0.22) for a unit increase in emotional intelligence (Table 3).

## 4. Discussion

Quality nursing care is essential to improve patient outcomes, develop the profession, and increase satisfaction for patients, families, and nurses. However, nurses are often undervalued and seen as subordinates to physicians. Evaluating nurses' clinical competence is a key component of assessing the quality of nursing care. However, there is a lack of recent and updated studies on this topic in Ethiopia.

The mean score of nurses' clinical competence was above average, which is consistent with studies conducted in Ethiopia [47], Iran [48], Taiwan [49], Finland [50], and Korea [24]. Nurses scored the highest mean in the clinical care, leadership, and interpersonal relations dimensions. They scored moderately in the legal/ethical practice and research aptitude/critical thinking dimensions and lowest in the professional development and teaching-coaching dimensions. This is also consistent with studies conducted elsewhere [44, 51]. A study from Ethiopia found that participants had higher competence scores on the legal/ethical dimension and lower competence scores on the teaching-coaching dimension [47]. However, a study from Iran found that 92.3% had good critical thinking and research attitude, 65.8% had moderate clinical care, and 73.5% had moderate leadership‏ [52]. Another study found that clinical competence was highest in areas relating to team collaboration and ethics and lowest in areas relating to professional development and direct clinical practice [53]. The discrepancy between these studies may be due to differences in the measurement of the constructs, study setting, health care system, and the nature of the clinical service delivery.

This study found that male nurse was significantly associated with higher clinical competence. This finding is inconsistent with studies conducted in Ethiopia [47] and Iran [54–56], which found no association between gender and clinical competence. The reason for this discrepancy may be that male nurses are more likely to perform clinical examinations and procedures than female nurses [57, 58], and they are also more confident in their abilities [59]. Additionally, the proportion of male nurses with more work experience was higher in this study.

Age was also found to be significantly associated with clinical competence in this study. However, the relationship was inverse meaning a unit increase in age decreases the clinical competence. This may be because older nurses are more resistant to change, they may be more fatigued, and they may not be as up-to-date on the latest clinical practices [60, 61]. The study found that marital status was a significant predictor of clinical competence. Single nurses had lower clinical competence than widowed nurses. This may be because widowed nurses have more experience and are more likely to have had to take on additional responsibilities after the death of their spouse. Additionally, widowed nurses may have more social support, which can help them to cope with the demands of the job.

A bachelor degree in nursing was significantly associated with the clinical competence. This was consistent with studies conducted in Ethiopia [47], Finland [62–64], Korea [65], and Taiwan [66]. Nurses with a bachelor's degree in nursing were expected to have better skills and knowledge because of the required competencies in the curriculum. This finding supports the current thinking that a degree adds value to competence [67]. Current position was also significantly associated with the clinical competence of the nurses. This was consistent with studies conducted in Korea [65], Taiwan [66], China [68], Iran [55], and Ethiopia [47]. Nurses in more senior positions, such as head nurses and clinical instructors, were more likely to have higher clinical competence. This may be because they have more experience and responsibility, and they are also more likely to have received additional training. Work experience showed a significant association with clinical competence. This was consistent with studies conducted in Ethiopia [47], Taiwan [66], Iran [31, 54], and Finland [62, 63]. Nurses with more work experience were more likely to have higher clinical competence. This may be because they have had more exposure to different patients and situations, and they have also had the opportunity to learn from their mistakes [47].

Studies from China [68] and Iran [55] found that working unit was a significant predictor of nurses' competence in practice. This study also supported this finding. However, nurses working in the medical and emergency unit had lower clinical competence than nurses working in other units. This may be because the proportion of diploma nurses in the medical and emergency unit was higher than in other units. Diploma nurses have lower levels of education and training than bachelor's degree nurses, and they are therefore less likely to have the skills and knowledge necessary for providing high-quality care in the medical and emergency units. This study also found that interest in the nursing profession was significantly associated with nurses' clinical competence. Nurses who were interested in the nursing profession were more likely to have higher clinical competence. This may be because they were more motivated to learn and develop their skills, and they were also more likely to be confident in their abilities.

Critical thinking disposition was found to be a predictor of clinical competence in studies conducted in Korea [24], Iran [25], and Taiwan [49]. This means that nurses who have a strong critical thinking disposition are more likely to have higher clinical competence. Critical thinking is the ability to think clearly and rationally, and it is an essential skill for nurses. Nurses who are able to think critically are better able to assess patients, make decisions, and provide safe and effective care. This study also reported that nurses who had higher levels of critical thinking disposition, such as intellectual integrity, creativity, challenge, open-mindedness, prudence, objectivity, truth-seeking, and inquisitiveness, also had higher levels of clinical competence. This suggests that critical thinking disposition is an important factor in clinical competence. Therefore, it is essential to develop nurses' critical thinking disposition through in-service education and short- and long-term training.

This study found that clinical self-efficacy had a significant effect on nurses' clinical competence. This is consistent with findings from studies conducted in Iran [69] and Taiwan [70]. Clinical competence and self-efficacy (another name for the nursing process) are highly correlated. Therefore, nurses who are competent in applying the nursing process to their patients in the clinical setting will also have high clinical competence, as these two concepts are two sides of the same coin. The study found that emotional intelligence is significantly associated with nurses' clinical competence. This is consistent with findings from studies conducted in Iran [71, 72]. Emotional intelligence is the ability to understand and manage one's own emotions as well as the emotions of others. It is essential for nurses, as they need to be able to build relationships with patients and colleagues and to manage stressful situations [73].

### 4.1. Implication of the Study

The importance of this study is paramount, as nurses' clinical competence is essential to maintaining the nursing profession as a profession that provides quality nursing care and improves patient outcomes. The findings of this study provide recent evidence of the status of nurses' clinical competence and the factors that influence it. This information is valuable for policymakers, program planners, and other scholars who are interested in conducting interventional studies to improve nurses' clinical competence. Additionally, the findings of this study can be used by scholars, nursing associations, and other concerned bodies who are working to improve the quality of nursing care.

### 4.2. Strength and Limitation of the Study

The main strengths of this study were its use of a multidimensional approach to measuring factors that affect clinical competence and its use of updated and validated tools to measure the outcome and its constructs. However, there were also some limitations to the study. The findings were subject to recall bias and social desirability bias, as the responses only included self-reported data. Additionally, it was difficult to show the temporal relationship between the factors that were studied and clinical competence. These limitations should be kept in mind when interpreting the findings of the study.

## 5. Conclusions

The overall level of clinical competence among nurses in this study was moderate. However, there was a significant variation among nurses. The following factors were significantly associated with clinical competence: gender, age, marital status, educational status, position, work experience, ward, interest in the nursing profession, critical thinking disposition, clinical self-efficacy, and emotional intelligence. The findings of this study add to the existing knowledge about clinical competence by providing new insights into the factors that contribute to it. The findings can also be used to reconcile the disparities between different clinical competence studies because they were assessed using a validated and reliable tool. This makes the findings more convincing and credible. Based on the findings of this study, there are a number of ways to improve the clinical competence of nurses. These include providing training and development opportunities that focus on critical thinking, clinical self-efficacy, and emotional intelligence; creating a supportive work environment that encourages nurses to take risks and learn from their mistakes; and monitoring the clinical competence of nurses on a regular basis and providing feedback and coaching as needed.

## Figures and Tables

**Table 1:** Background and professional characteristics of the nurses in hospitals of Gamo Zone, Southern Ethiopia, 2022 (*n* *=* 404).

Variables	Frequency	Percentage
*Gender*		
Female	261	64.6
Male	143	35.4
*Age (in a year)*		
25–30	149	36.9
31–35	133	32.9
36–40	86	21.3
≥41	36	8.9
*Marital status*		
Single	124	30.7
Married	260	64.4
Widowed	20	5.0
*Religion*		
Orthodox	240	59.4
Protestant	164	40.6
*Work experience (in a year)*		
1–5	89	22.0
6–10	125	30.9
11–15	136	33.7
≥16	54	13.4
*Qualification*		
BSc	252	62.4
Diploma	152	37.6
*Current working unit*		
Medical ward	88	21.8
Surgical ward	115	28.5
Pediatric ward	73	18.1
NICU	58	14.4
Emergency unit	40	9.9
OR	30	7.4
*Training in nursing care*		
Yes	164	40.6
No	240	59.4
*Interest in the nursing profession*		
Poor	39	9.7
Fair	65	16.1
Good	300	74.3

**Table 2:** Simple linear regression for the factors associated with the clinical competence of nurses in hospitals of Gamo Zone, Southern Ethiopia, 2022 (*n* *=* 404).

Variables	Standardized coefficients, *β*	*t*	*P* value	95% CI of *β*	*R* ^2^	Adjusted *R*^2^
Gender (male)	0.25	5.18	<0.001^*∗*^	(0.16, 0.35)	0.06	0.06
Age	−0.27	−5.63	<0.001^*∗*^	(−0.37, −0.18)	0.07	0.07
Marital status						
Single	0.03	0.58	0.56	(0.56, −0.07)	0.001	−0.002
Married	0.04	0.83	0.41	(−0.06, 0.14)	0.002	−0.001
Qualification (BSc)	0.37	7.87	<0.001^*∗*^	(0.27, 0.46)	0.13	0.13
Position/title						
Focal person	0.10	2.02	0.04^*∗*^	(0.003, 0.19)	0.01	0.01
Nursing director	0.10	2.05	0.04^*∗*^	(0.004, 0.19)	0.01	0.01
Work experience	−0.07	−1.34	0.18	(−0.17, 0.03)	0.004	0.002
Ward/unit						
Medical ward	−0.25	−5.19	<0.001^*∗*^	(−0.35, −0.16)	0.06	0.06
Surgical ward	0.43	9.59	<0.001^*∗*^	(0.34, 0.52)	0.19	0.18
Pediatric ward	−0.05	−1.02	0.31	(−0.15, 0.05)	0.003	0.00
NICU	−0.001	−0.02	0.98	(−0.09, 0.09)	0.00	−0.002
Emergency unit	−0.17	−3.55	<0.001^*∗*^	(−0.27, −0.07)	0.03	0.03
Training in nursing care	0.25	5.07	<0.001^*∗*^	(0.15, 0.34)	0.06	0.06
Interest in the nursing profession						
Fair	0.12	2.38	0.02^*∗*^	(0.02, 0.22)	0.01	0.01
Good	0.07	1.37	0.17	(−0.03, 0.17)	0.005	0.002
Practice environment	0.49	11.39	<0.001^*∗*^	(0.41, 0.58)	0.24	0.24
Critical thinking disposition	0.47	10.54	<0.001^*∗*^	(0.38, 0.55)	0.22	0.22
Professional quality of life	0.51	11.73	<0.001^*∗*^	(0.42, 0.59)	0.26	0.25
Clinical self-efficacy	0.30	6.37	<0.001^*∗*^	(0.21, 0.39)	0.09	0.09
Personal traits	0.34	7.13	<0.001^*∗*^	(0.24, 0.43)	0.11	0.11
Emotional intelligence	0.39	8.57	<0.001^*∗*^	(0.30, 0.48)	0.15	0.15

^*∗*^Significant at *P* value <0.05.

**Table 3:** Multiple linear regression for the factors associated with the clinical competence of nurses in hospitals of Gamo Zone, Southern Ethiopia, 2022 (*n* *=* 404).

Variables	Standardized coefficients, *β*	*t*	*P* value	95% CI of *β*
Gender (male)	0.19	4.07	<0.001^*∗*^	(0.09, 0.28)
Age	−0.66	−8.72	<0.001^*∗*^	(−0.81, −0.51)
Marital status				
Single	−0.31	−2.69	0.007^*∗*^	(−0.54, −0.08)
Married	−0.16	−1.55	0.12	(−0.37, 0.04)
Qualification (BSc)	0.26	6.91	<0.001^*∗*^	(0.18, 0.33)
Position/title				
Focal person	0.12	3.24	0.001^*∗*^	(0.05, 0.19)
Nursing director	0.02	0.57	0.57	(−0.05, 0.09)
Work experience	0.43	6.38	<0.001^*∗*^	(0.29, 0.56)
Ward/unit				
Medical ward	−0.29	−5.26	<0.001^*∗*^	(−0.39, −0.18)
Surgical ward	−0.07	−1.02	0.31	(−0.19, 0.06)
Pediatric ward	−0.07	−1.21	0.23	(−0.18, 0.04)
NICU	−0.06	−1.01	0.31	(−0.18, 0.06)
Emergency unit	−0.15	−3.34	0.001^*∗*^	(−0.24, −0.06)
Training in nursing care	0.05	1.09	0.28	(−0.04, 0.13)
Interest in the nursing profession				
Fair	0.25	4.07	<0.001^*∗*^	(0.13, 0.37)
Good	0.17	2.83	0.005^*∗*^	(0.05, 0.28)
Practice environment	0.10	1.60	0.11	(−0.02, 0.23)
Critical thinking disposition	0.35	7.97	<0.001^*∗*^	(0.27, 0.44)
Professional quality of life	−0.04	−0.86	0.39	(−0.13, 0.05)
Clinical self-efficacy	0.11	2.82	0.005^*∗*^	(0.03, 0.19)
Personal traits	0.01	0.38	0.71	(−0.06, 0.09)
Emotional intelligence	0.14	3.61	<0.001^*∗*^	(0.06, 0.22)

^*∗*^Significant at *P* value <0.05, *R*^2^ = 0.70, and adjusted *R*^2^ = 0.69.

## Data Availability

The data used to support the findings of this study are available from the corresponding author upon request.

## Supplementary Materials

Supplementary Materials Supplementary file 1: English version questionnaire (Word).
