# Supplementary material for: Clinical Competence of Nurses and the Associated Factors in Public Hospitals of Gamo Zone, Southern Ethiopia: A Cross-Sectional Study
Source: Nurs Res Pract. 2023 Sep 26;2023:9656636. doi: 10.1155/2023/9656636 (PMC10547574; doi:10.1155/2023/9656636)
Supplement: Supplementary Materials — Supplementary file 1: English version questionnaire (Word). [file 9656636.f1.docx]

Questionnaire developed to assess the clinical competence of nurses and its associated factors in hospitals of Gamo zone, southern Ethiopia, 2022

| **SNo** | **Questions** | **Response** | **Skip** |
| --- | --- | --- | --- |
| **Part I: Socio-demographic and professional-related characteristics** | | | |
|  | Sex | 1. Male 2. Female |  |
|  | Age | **_________(**in year) |  |
|  | Religion | 1. Orthodox 2. Protestant 3. Muslim 4. Catholic 5. Other_____________ |  |
|  | Marital status | 1. Single 2. Married 3. Divorced 4. Widowed 5. Separated due to work |  |
|  | Educational level | 1. Diploma 2. BSc 3. MSc 4. Other_________________ |  |
|  | Position/title | 1. No (staff nurse) 2. Ward leader/focal person 3. Nurse manager/nursing director 4. Matron 5. Other________________ |  |
|  | Experience working as a nurse | _____________(in year) |  |
|  | Current working unit | 1. Medical ward 2. Surgical ward 3. Pediatric ward 4. NICU 5. Emergency unit 6. OR |  |
|  | Ant training in nurses care | 1. Yes 2. No | If no  Go to #111 |
|  | If yes, for the above question | Specify_______________ |  |
|  | Interest in nursing profession | 1. Poor 2. Fair 3. Good |  |

| **Part II: Clinical competency inventory for registered nurse (CIRN): 5 points Likert scale** | | | | | | | |
| --- | --- | --- | --- | --- | --- | --- | --- |
| **SNo** | **Characteristics** | **0** | **1** | **2** | **3** | **4** | **Remark** |
| ***Clinical care*** | | | | | | | |
|  | Provides culturally-sensitive care |  |  |  |  |  |  |
|  | Identifies and includes immediate patient needs in the plan of nursing care |  |  |  |  |  |  |
|  | Give emotional support to families |  |  |  |  |  |  |
|  | Assesses all health dimensions of client, i.e. physical, psycho-social, spiritual aspects |  |  |  |  |  |  |
|  | Develops a nursing care plan for a specific patient based on a primary and secondary data base |  |  |  |  |  |  |
|  | Delivers accurate, comprehensive and effective nursing in accordance with the plan |  |  |  |  |  |  |
|  | Involves the patient and family in the planning and implementation of care |  |  |  |  |  |  |
|  | Utilizes technological advances to improve nursing and health care |  |  |  |  |  |  |
|  | Detects and document significant changes in a patient’s condition |  |  |  |  |  |  |
|  | Evaluates results of nursing care interventions |  |  |  |  |  |  |
| ***Leadership*** | | | | | | | |
|  | Recognizes other’s contribution and achievement |  |  |  |  |  |  |
|  | Accepts and uses constructive criticism |  |  |  |  |  |  |
|  | Delegates responsibility for care based on assessment of abilities of individuals |  |  |  |  |  |  |
|  | Gets group approval in important matters before acting |  |  |  |  |  |  |
|  | Acts to develop an atmosphere for teamwork and cooperation |  |  |  |  |  |  |
|  | Promotes cooperation, trust, and open exchange of ideas |  |  |  |  |  |  |
|  | Resolves conflict in a positive way |  |  |  |  |  |  |
|  | Identifies and understands others’ personal strengths and weaknesses |  |  |  |  |  |  |
|  | Coordinates the relation between nurses and all related personnel |  |  |  |  |  |  |
| ***Interpersonal relations*** | | | | | | | |
|  | Adjusts actions in relation to other’s actions |  |  |  |  |  |  |
|  | Cooperates with other care providers solving to meet patient needs |  |  |  |  |  |  |
|  | Communicates facts, ideas and feelings to other health team member verbally |  |  |  |  |  |  |
|  | Builds trust by keeping word, commitments, and promises |  |  |  |  |  |  |
|  | Acknowledges the differences in beliefs and cultural practices of individuals/groups |  |  |  |  |  |  |
|  | Shows willingness to share workload when needed |  |  |  |  |  |  |
|  | Expresses facts and thoughts in writing in a clear and organized way |  |  |  |  |  |  |
|  | Expresses disagreements in a constructive manner |  |  |  |  |  |  |
| ***Legal/ethical practice*** | | | | | | | |
|  | Carries out nursing practice according to legal requirements and organizational policy |  |  |  |  |  |  |
|  | Functions in accordance with legislative and common law affecting nursing practice |  |  |  |  |  |  |
|  | Takes responsibility for one’s own performance |  |  |  |  |  |  |
|  | Serves as an advocate for the rights of clients or groups |  |  |  |  |  |  |
|  | Respects the patient’s/client’s right to privacy |  |  |  |  |  |  |
|  | Ensures confidentiality and security of written and verbal information acquired in a professional capacity |  |  |  |  |  |  |
|  | Reports all perceived malpractice incidents to responsible persons |  |  |  |  |  |  |
|  | Respects the patient’s/client’s right to choice and self-determination in nursing and health care |  |  |  |  |  |  |
| ***Professional development*** | | | | | | | |
|  | Understands role of professional organizations and actively participates in them |  |  |  |  |  |  |
|  | Displays self-direction in personal development |  |  |  |  |  |  |
|  | Uses learning opportunities for ongoing personal and professional growth |  |  |  |  |  |  |
|  | Recognizes own learning needs |  |  |  |  |  |  |
|  | Demonstrates self-awareness of personal limitations & strengths |  |  |  |  |  |  |
|  | Understands relevant and current information concerning health care system |  |  |  |  |  |  |
| ***Teaching-coaching*** | | | | | | | |
|  | Identifies learning needs of others including patients, families, and junior nurses |  |  |  |  |  |  |
|  | Coaches junior nurses to meet both the task needs and their developmental needs |  |  |  |  |  |  |
|  | Takes up the preceptor role to support new nurses in adapting to a new working environment |  |  |  |  |  |  |
|  | Initiates the appropriate orientation programs for new nurses |  |  |  |  |  |  |
|  | Uses opportunities for patient teaching when they arise |  |  |  |  |  |  |
|  | Develops an explicit teaching strategy to teach patients and families |  |  |  |  |  |  |
| ***Research aptitude/critical thinking*** | | | | | | | |
|  | Defends decisions using scientific knowledge principles |  |  |  |  |  |  |
|  | Identifies priority risk in clinical situations |  |  |  |  |  |  |
|  | Makes decisions that reflect both knowledge of facts and good judgment |  |  |  |  |  |  |
|  | Figures out more than one way to solve confronting clinical problems |  |  |  |  |  |  |
|  | Assists in the clinical research data collection |  |  |  |  |  |  |
|  | Integrates pertinent data from multiple sources |  |  |  |  |  |  |
|  | Uses different ways to search for information |  |  |  |  |  |  |
|  | Incorporates relevant research findings into nursing practice |  |  |  |  |  |  |

***Remark: incompetency (0), low competency (1), moderate competency (2), high or sufficient competency (3), and very high competency (4)***

| **Part III: Nursing Work Index Revised (NWI-R) instrument to measure practice environment: 4 points Likert scale** | | | | | | |
| --- | --- | --- | --- | --- | --- | --- |
| **SNo** | **Characteristics** | **1** | **2** | **3** | **4** | **Remark** |
| ***Adequate staffing and resources*** | | | | | | |
|  | Adequate support services allow me to spend time with my patients |  |  |  |  |  |
|  | Enough time and opportunity to discuss patient care problems |  |  |  |  |  |
|  | Enough registered nurses on staff to provide quality patient care |  |  |  |  |  |
|  | Enough staff to get the work done |  |  |  |  |  |
| ***Autonomy*** | | | | | | |
|  | Freedom to make important patient care and work decisions |  |  |  |  |  |
|  | Managerial staffs that are supportive of nurses |  |  |  |  |  |
|  | Nursing controls its own practice |  |  |  |  |  |
|  | A ward leader who is a good manager and leader |  |  |  |  |  |
|  | A nurse manager backs up the nursing staff in decision making, even if it conflicts with a doctor |  |  |  |  |  |
| ***Nurse-physician collaboration*** | | | | | | |
|  | Doctors and nurses have good working relationships |  |  |  |  |  |
|  | Much teamwork between doctors and nurses |  |  |  |  |  |
|  | Collaboration (joint practice) between doctors and nurses |  |  |  |  |  |

***Remark: strongly disagree (1), disagree (2), agree (3), strongly agree (4)***

| **Part IV: Critical disposition scales: 5 points Likert scale** | | | | | | | |
| --- | --- | --- | --- | --- | --- | --- | --- |
| **SNo** | **Characteristics** | **1** | **2** | **3** | **4** | **5** | **Remark** |
| ***Intellectual integrity*** | | | | | | | |
|  | Before judging a problem, I overall take the related information into account |  |  |  |  |  |  |
|  | When I approach a problem, I consider the whole situation of the problem such as relationship or background |  |  |  |  |  |  |
|  | When I solve or judge a problem, I utilize a collections of data by organizing it systematically |  |  |  |  |  |  |
|  | When I confront a problem, first of all, I try to grasp the whole picture of it |  |  |  |  |  |  |
|  | When I confront a problem, I consider solving it from many different perspectives |  |  |  |  |  |  |
|  | When I decide on something, I make a decision based on reliable and sufficient data |  |  |  |  |  |  |
| ***Creativity*** | | | | | | | |
|  | I like to develop an original idea |  |  |  |  |  |  |
|  | I prefer to think differently from others |  |  |  |  |  |  |
|  | I usually come up with an idea that other people don't have |  |  |  |  |  |  |
|  | I apply a different method than I tried before to solve a problem |  |  |  |  |  |  |
| ***Challenge*** | | | | | | | |
|  | I hesitate to start on something that looks difficult |  |  |  |  |  |  |
|  | I easily give up on the way of doing a project |  |  |  |  |  |  |
|  | I give up a debate when it does not come to a conclusion quickly |  |  |  |  |  |  |
|  | I tend to follow what others do |  |  |  |  |  |  |
|  | I tend not to come up with a conclusion |  |  |  |  |  |  |
|  | I have a tendency not taking a change well |  |  |  |  |  |  |
| ***Open-mindedness*** | | | | | | | |
|  | I willingly accept a criticism on my opinion |  |  |  |  |  |  |
|  | I like to listen to other people's opinion on an argument |  |  |  |  |  |  |
|  | I turn my mistake into an opportunity to learn |  |  |  |  |  |  |
| ***Prudence*** | | | | | | | |
|  | I tend to make a decision hastily without considering a matter carefully |  |  |  |  |  |  |
|  | When I am questioned, I think twice before I give my answer |  |  |  |  |  |  |
|  | I tend to act rashly and carelessly when I face a difficulty |  |  |  |  |  |  |
|  | Before I decide on something, I think ahead the advantages and shortcomings of the result of it |  |  |  |  |  |  |
| ***Objectivity*** | | | | | | | |
|  | I have a reputation of being a rational person |  |  |  |  |  |  |
|  | When I judge a matter, I judge objectively |  |  |  |  |  |  |
|  | I'm hard for me to be fair when I discuss a matter related to me directly |  |  |  |  |  |  |
|  | Ordinarily, I analyze which is right or wrong |  |  |  |  |  |  |
| ***Truth seeking*** | | | | | | | |
|  | When I work on something, I repeatedly appraise the matter |  |  |  |  |  |  |
|  | Although the direction of a project is set, I continue to ponder about it to make a better result |  |  |  |  |  |  |
|  | I continually evaluate whether my thought is right or not |  |  |  |  |  |  |
| ***Inquisitiveness*** | | | | | | | |
|  | When something is happened, I am curious about the process of it |  |  |  |  |  |  |
|  | When I have a question, I try to get the answer |  |  |  |  |  |  |
|  | I enjoy trying to solve a complicated problem |  |  |  |  |  |  |
|  | I continually look for pieces of information related to solving a problem |  |  |  |  |  |  |
|  | When I see the world, I see it with a questioning mind |  |  |  |  |  |  |

***Remark: strongly disagree (1), disagree (2), neutral (3), agree (4), and strongly agree (5)***

| **Part V: Professional quality of life (ProQOL): 5 points Likert scale** | | | | | | | |
| --- | --- | --- | --- | --- | --- | --- | --- |
| **SNo** | **Characteristics** | **1** | **2** | **3** | **4** | **5** | **Remark** |
| ***Compassion satisfaction*** | | | | | | | |
|  | I get satisfaction from being able to [help] people |  |  |  |  |  |  |
|  | I feel invigorated after working with those I [help] |  |  |  |  |  |  |
|  | I like my work as a [helper] |  |  |  |  |  |  |
|  | I am pleased with how I am able to keep up with [helping] techniques and protocols |  |  |  |  |  |  |
|  | My work makes me feel satisfied |  |  |  |  |  |  |
|  | I have happy thoughts and feelings about those I [help] and how I could help them |  |  |  |  |  |  |
|  | I believe I can make a difference through my work |  |  |  |  |  |  |
|  | I am proud of what I can do to [help] |  |  |  |  |  |  |
|  | I have thoughts that I am a "success" as a [helper] |  |  |  |  |  |  |
|  | I am happy that I chose to do this work |  |  |  |  |  |  |
| ***Burnout*** | | | | | | | |
|  | I am happy* |  |  |  |  |  |  |
|  | I feel connected to others* |  |  |  |  |  |  |
|  | I am not as productive at work because I am losing sleep over traumatic experiences of a person I [help] |  |  |  |  |  |  |
|  | I feel trapped by my job as a [helper] |  |  |  |  |  |  |
|  | I have beliefs that sustain me* |  |  |  |  |  |  |
|  | I am the person I always wanted to be* |  |  |  |  |  |  |
|  | I feel worn out because of my work as a [helper] |  |  |  |  |  |  |
|  | I feel overwhelmed because my case [work] load seems endless |  |  |  |  |  |  |
|  | I feel "bogged down" by the system |  |  |  |  |  |  |
|  | I am a very caring person* |  |  |  |  |  |  |
| ***Secondary traumatic stress*** | | | | | | | |
|  | I am preoccupied with more than one person I [help] |  |  |  |  |  |  |
|  | I jump or am startled by unexpected sounds |  |  |  |  |  |  |
|  | I find it difficult to separate my personal life from my life as a [helper] |  |  |  |  |  |  |
|  | I think that I might have been affected by the traumatic stress of those I [help] |  |  |  |  |  |  |
|  | Because of my [helping], I have felt "on edge" about various things |  |  |  |  |  |  |
|  | I feel depressed because of the traumatic experiences of the people I [help] |  |  |  |  |  |  |
|  | I feel as though I am experiencing the trauma of someone I have [helped] |  |  |  |  |  |  |
|  | I avoid certain activities or situations because they remind me of frightening experiences of the  people I [help] |  |  |  |  |  |  |
|  | As a result of my [helping], I have intrusive, frightening thoughts |  |  |  |  |  |  |
|  | I can't recall important parts of my work with trauma victims |  |  |  |  |  |  |

***Remark: never (1), rarely (2), sometimes (3), often (4), and very often (5), and*** *** ***Reverse scored***

| **Part VI: Self-Efficacy in Clinical Performance (SECP) instrument: 4 points Likert scale** | | | | | | |
| --- | --- | --- | --- | --- | --- | --- |
| **SNo** | **Characteristics** | **0** | **1** | **2** | **3** | **Remark** |
| ***Assessment*** | | | | | | |
|  | Collect significance data by physical assessment |  |  |  |  |  |
|  | Collect relevant data by taking patient’s history |  |  |  |  |  |
|  | Collect data by restoration of patient’s and mine energy |  |  |  |  |  |
|  | Collect data by organizing time available |  |  |  |  |  |
|  | Collect objective data related to patient health condition |  |  |  |  |  |
|  | Collect subjective data related to patient health condition |  |  |  |  |  |
|  | See relationships among pieces of data that were collected from variety of sources |  |  |  |  |  |
|  | Document collecting data based on patient health condition |  |  |  |  |  |
|  | Analyse collecting data based on patient health condition |  |  |  |  |  |
|  | Identify patient’s strengths in the care process |  |  |  |  |  |
|  | Identify patient’s health concerns in the care process |  |  |  |  |  |
|  | Set priority of patient’s problems based on patient health condition |  |  |  |  |  |
| ***Diagnosis*** | | | | | | |
|  | Formulate nursing diagnosis based on collecting data |  |  |  |  |  |
|  | Formulate nursing diagnosis based on contributing factors of patient’s problems |  |  |  |  |  |
|  | Arrange nursing diagnosis based on setting priorities |  |  |  |  |  |
| ***Planning*** | | | | | | |
|  | Formulate overall goals of patients care plan |  |  |  |  |  |
|  | Formulate short-term goals of patients care plan |  |  |  |  |  |
|  | Formulate long-term goals of patients care plan |  |  |  |  |  |
|  | Formulate measurable outcomes based on goals |  |  |  |  |  |
|  | Develop patient’s daily care plan based on goals |  |  |  |  |  |
|  | Develop patient’s daily care plan based on setting priorities |  |  |  |  |  |
| ***Implementation*** | | | | | | |
|  | Follow the direction of patient’s care plan to achieve setting goals |  |  |  |  |  |
|  | Take care of patient based on setting priorities |  |  |  |  |  |
|  | Carry out patient’s care plan based on available resources |  |  |  |  |  |
|  | Explain each nursing intervention to patient or family members before carrying it out |  |  |  |  |  |
|  | Collaborate with patient or family members in implementation of daily care plan |  |  |  |  |  |
|  | Make decision based on my previous experiences in similar situation |  |  |  |  |  |
|  | Seek help of mentor or staff in difficult situations |  |  |  |  |  |
|  | Improve my skills based on mentor and staff feedback |  |  |  |  |  |
|  | Design teaching strategies for discharge of patient |  |  |  |  |  |
|  | Document and report daily clinical work |  |  |  |  |  |
| ***Evaluation*** | | | | | | |
|  | Evaluate whether patient’s desired results were achieved |  |  |  |  |  |
|  | Evaluate how nursing interventions are carried out |  |  |  |  |  |
|  | Find the point of breakdown in the steps of nursing process |  |  |  |  |  |
|  | Decide about continuing or modifying care plan based on patient’s prognosis |  |  |  |  |  |
|  | Decide about changing outcome based on patient’s prognosis |  |  |  |  |  |
|  | Decide about adjusting setting priorities based on patient’s prognosis |  |  |  |  |  |

***Remark: never (0), rarely (1), sometimes (2), and quite always (3)***

| **Part VII: Big Five Inventory‐10 (BFI‐10) to measure personal traits: 5 points Likert scale** | | | | | | | |
| --- | --- | --- | --- | --- | --- | --- | --- |
| **Instructions: How well do the following statements describe your personality?** | | | | | | | |
| **SNo** | **Characteristics** | **1** | **2** | **3** | **4** | **5** | **Remark** |
|  | I see myself as someone who is reserved* |  |  |  |  |  |  |
|  | I see myself as someone who is generally trusting |  |  |  |  |  |  |
|  | I see myself as someone who tends to be lazy* |  |  |  |  |  |  |
|  | I see myself as someone who is relaxed, handles stress well* |  |  |  |  |  |  |
|  | I see myself as someone who has few artistic interests* |  |  |  |  |  |  |
|  | I see myself as someone who is outgoing, sociable |  |  |  |  |  |  |
|  | I see myself as someone who tends to find fault with others* |  |  |  |  |  |  |
|  | I see myself as someone who does a thorough job |  |  |  |  |  |  |
|  | I see myself as someone who gets nervous easily |  |  |  |  |  |  |
|  | I see myself as someone who has an active imagination |  |  |  |  |  |  |

***Remark: strongly disagree (1), disagree (2), neutral (3), agree (4), and strongly agree (5), and*** ******Reverse scored***

| **Part VIII: The Schutte Self Report Emotional Intelligence Test (SSEIT) to measure emotional intelligence: 5 points Likert scale** | | | | | | | |
| --- | --- | --- | --- | --- | --- | --- | --- |
| **SNo** | **Characteristics** | **1** | **2** | **3** | **4** | **5** | **Remark** |
|  | I know when to speak about my personal problems to others |  |  |  |  |  |  |
|  | When I am faced with obstacles, I remember times I faced similar obstacles and overcame them |  |  |  |  |  |  |
|  | I expect that I will do well on most things I try |  |  |  |  |  |  |
|  | Other people find it easy to confide in me |  |  |  |  |  |  |
|  | I find it hard to understand the non-verbal messages of other people* |  |  |  |  |  |  |
|  | Some of the major events of my life have led me to re-evaluate what is important and not important |  |  |  |  |  |  |
|  | When my mood changes, I see new possibilities |  |  |  |  |  |  |
|  | Emotions are one of the things that make my life worth living |  |  |  |  |  |  |
|  | I am aware of my emotions as I experience them |  |  |  |  |  |  |
|  | I expect good things to happen |  |  |  |  |  |  |
|  | I like to share my emotions with others |  |  |  |  |  |  |
|  | When I experience a positive emotion, I know how to make it last |  |  |  |  |  |  |
|  | I arrange events others enjoy |  |  |  |  |  |  |
|  | I seek out activities that make me happy |  |  |  |  |  |  |
|  | I am aware of the non-verbal messages I send to others |  |  |  |  |  |  |
|  | I present myself in a way that makes a good impression on others |  |  |  |  |  |  |
|  | When I am in a positive mood, solving problems is easy for me |  |  |  |  |  |  |
|  | By looking at their facial expressions, I recognize the emotions people are experiencing |  |  |  |  |  |  |
|  | I know why my emotions change |  |  |  |  |  |  |
|  | When I am in a positive mood, I am able to come up with new ideas |  |  |  |  |  |  |
|  | I have control over my emotions |  |  |  |  |  |  |
|  | I easily recognize my emotions as I experience them |  |  |  |  |  |  |
|  | I motivate myself by imagining a good outcome to tasks I take on |  |  |  |  |  |  |
|  | I compliment others when they have done something well |  |  |  |  |  |  |
|  | I am aware of the non-verbal messages other people send |  |  |  |  |  |  |
|  | When another person tells me about an important event in his or her life, I almost feel as  though I have experienced this event myself |  |  |  |  |  |  |
|  | When I feel a change in emotions, I tend to come up with new ideas |  |  |  |  |  |  |
|  | When I am faced with a challenge, I give up because I believe I will fail* |  |  |  |  |  |  |
|  | I know what other people are feeling just by looking at them |  |  |  |  |  |  |
|  | I help other people feel better when they are down |  |  |  |  |  |  |
|  | I use good moods to help myself keep trying in the face of obstacles |  |  |  |  |  |  |
|  | I can tell how people are feeling by listening to the tone of their voice |  |  |  |  |  |  |
|  | It is difficult for me to understand why people feel the way they do* |  |  |  |  |  |  |

***Remark: strongly disagree (1), disagree (2), neutral (3), agree (4), and strongly agree (5)*** ***and*** ******Reverse scored***
